# Diagnostic Challenges of Short Stature and Growth Hormone Insufficiency Across Different Genetic Etiologies

**DOI:** 10.3390/biomedicines13081937

**Published:** 2025-08-08

**Authors:** Federica Arzilli, Giulia De Fortuna, Ignazio Cammisa, Luca Vagnozzi, Giorgio Sodero, Donato Rigante, Clelia Cipolla

**Affiliations:** 1 Department of Life Sciences and Public Health, Fondazione Policlinico Universitario A. Gemelli IRCCS, 00168 Rome, Italyignazio.cammisa01@icatt.it (I.C.); donato.rigante@unicatt.it (D.R.); clelia.cipolla@policlinicogemelli.it (C.C.); 2 Pediatric Department, Perrino Hospital, 72100 Brindisi, Italy; 3 Pediatric Endocrinology Unit, Perrino Hospital, 72100 Brindisi, Italy; 4 Department of Pediatrics, Università Cattolica del Sacro Cuore, 00168 Rome, Italy

**Keywords:** short stature, children, growth, growth hormone deficiency, genetics, personalized medicine, innovative biotechnologies

## Abstract

**Background**: Recent advances in genetic research have significantly expanded our understanding of the molecular bases of growth hormone deficiency (GHD), and numerous genes have been identified as impacting final stature through isolated or combined abnormalities of growth hormone (GH), GH insensitivity, and insulin growth factor-1 (IGF-I) resistance. **Objective**: This review summarizes the current knowledge on the genetic causes of GHD in the context of pediatric short stature, emphasizing the role of next-generation sequencing technologies in real-life clinical practice and the potential impact of genetic diagnosis over therapeutic decisions regarding GH replacement therapy. **Materials and methods**: Articles from PubMed up to April 2025 dealing with GHD were retrieved and analyzed, focusing on genes influencing the GH pathway and stunted growth, with focused attention on relevant molecular and clinical studies. **Results**: Our analysis, besides cataloguing well-established and novel contributors to growth failure among genes associated with the GH–IGF1 axis, also emphasizes the crucial role of genetic testing and strategies that should be used to maximize the likelihood of identifying a specific genetic etiology, such as prioritizing genetic tests when a monogenic cause is strongly suspected or when there are peculiar clinical features that could be linked to specific genetic conditions. **Conclusions**: We have highlighted the most recent genetic etiologies of short stature related to GHD, providing an updated framework that is expected to be helpful in the diagnostic and therapeutic management of individuals with mutations related to the GH-IGF1 axis.

## 1. Introduction

Short stature, defined as a height of at least two standard deviations (SD) below the normal mean value for age and sex in a reference population, is a very frequent referral assessment request in pediatric endocrinology. While many children with short stature are diagnosed as displaying normal variant patterns of growth, a subset may reveal underlying genetic disorders leading to idiopathic short stature (ISS) [1,2,3]. Children with ISS primarily fall into two main subgroups: (a) familial short stature (FSS), which involves healthy growth and development in children with a family history of short stature, and (b) constitutional delay in growth and puberty (CDGP), in which children exhibit delayed bone maturation and later onset of puberty, but ultimately attain an adult height within their genetic target range [1,4,5]. In both cases, children have normal body proportions and lack any detectable systemic, endocrine, nutritional, or chromosomal abnormalities [1,5]. Furthermore, it is well-established that adult stature is primarily determined by genetic factors, as demonstrated by genome-wide association studies that have identified over 500 genetic loci associated with human height, as well as by studies investigating monogenic disorders, which show that rare genetic variants also play a significant role in determining stature, in either homozygosity or heterozygosity, in non-syndromic individuals [6,7]. Among endocrine causes, growth hormone deficiency (GHD) represents one of the most clinically significant, resulting from inadequate production or secretion of the growth hormone (GH), bringing about impaired linear growth and, if untreated, leading to suboptimal final height in adulthood.

GHD is one of the most important sources of short stature, being characterized by subnormal release of GH from the anterior pituitary [6,7,8,9,10]. This hormone normally activates different complex pathways by binding to GH receptors on bone tissue, liver, muscles, and fat and by stimulating the liver to produce insulin-like growth factor 1 (IGF-1). Both GH and IGF-1 act on the growth plate regulating chondrocyte differentiation and proliferation [9]. Therefore, GHD drives impaired linear growth and, left untreated, also results in an inadequate adult final height [8,9]. Although the typical phenotype of GHD includes short stature with height ≤ −2 SD, frontal bossing, midfacial hypoplasia, and truncal adiposity, it can also present as a delay in the growth velocity or isolated short stature; the differential diagnosis of these conditions is tricky, as growth delay is also common in patients undergoing pharmacological therapies, such as glucocorticoids, or in those receiving long-term chronic treatments [8].

Advances in genetic testing have also improved our knowledge of the genetic bedrock of stunted growth in GHD, identifying monogenic forms of isolated GHD as well as genes associated with syndromic forms of GHD [3,6]. The genes identified to date act through three distinct mechanisms: GH deficiency (either isolated or combined with other hypothalamic–pituitary hormone deficiencies), GH insensitivity, and IGF-I resistance [11,12,13]. Understanding the genetic background of GHD not only facilitates a more accurate diagnosis and personalizes the treatment strategy, but also provides valuable insights into the pituitary organogenesis and endocrine regulation. Moreover, genetic testing is increasingly recognized as a key-tool, particularly in cases with GHD and no identifiable structural abnormalities or when there is a positive family history. The main aim of this review is to summarize the currently known genetic causes of GHD in the context of short stature, highlighting the importance of next-generation diagnostic approaches and the impact of these discoveries on GH replacement therapy.

## 2. Search Strategy

The research was conducted using the PubMed database in order to identify all articles related to genes involved in the GH pathway implicated at different levels in short stature, up to April 2025. Specifically, three main previous reviews were helpful in analyzing all the current medical literature related to genetic disorders causing short stature. Furthermore, specific articles were found in relationship with all the best-known genes affecting the GH pathway, making a step-by-step analysis that included also the pattern of transmission and clinical phenotype for the majority of the genetic abnormalities studied. This research was carried out considering both a manual screening of the references of the three key articles and also using free-text terms. In this last case, the specific names used to retrieve papers were “genetic causes or diseases or diagnosis” AND “short stature”; for the combined pituitary hormone deficiency, the combination of “pituitary disorders” and “hypopituitarism” was also applied, while for IGHD, we included the association of “GH deficiency or pathway”, “disorders of growth hormone”, AND “GH insensitivity”.

All research articles were considered and analyzed, focusing on genetic, molecular and clinical studies related to alterations in the GH pathway, and selected for their methodological relevance. It was decided to include only articles written in English and published in peer-reviewed journals. GenAI has been used to assist in the study design in relationship with disorders of the pituitary gland, in order to create a clearer distinction between genes involved in pituitary ontogenesis and the ones with an important role in pituitary somatotroph differentiation. Moreover, GenAI was used to structure tables and sum up the key elements of the genes described in this review. The goal of the whole selection process was to provide a comprehensive updated assessment of all current medical literature articles regarding this field of pediatric endocrinology.

## 3. Main Findings

### 3.1. Genetic Testing in Short Stature

While a child’s short stature might sometimes stem from the combined effect of multiple genetic variants, each with a small individual impact—a pattern known as oligogenic or polygenic, often seen in FSS—current genetic testing primarily aims to identify single causative genetic variants, referred to as monogenic causes of short stature. Therefore, a scrupulous selection of patients for genetic evaluation is crucial. To maximize the likelihood of identifying specific genetic etiologies, testing should be prioritized for individuals in whom a monogenic cause of their short stature is strongly suspected. Without this careful selection, genetic testing may not reveal the underlying reason for the child’s reduced growth [3]. Therefore, genetic testing should be considered in children with short stature when other clinical features or growth patterns ideally suggest an underlying genetic explanation. These indications can include a significant deviation from the familial height potential, presence of dysmorphic features, developmental delay, or family history of genetic conditions associated with short stature [11]. On the other hand, performing genetic testing should also be considered if all biochemical studies show negative results and there is no relevant phenotype to flag [3]. Several types of genetic tests are available, ranging from general karyotyping to targeted single-gene testing of specific diseases like Turner or Noonan syndrome [14,15]; broader approaches such as chromosomal microarray analysis to detect copy number variations [16]; or more comprehensive methods like whole-exome or whole-genome sequencing for identifying rare genetic variants [10]. The results of genetic testing significantly impact the follow-up and management of these children, for example, they could offer the opportunity to predict a patient’s response to GH treatment and provide the opportunity for family genetic counseling [3,11].

### 3.2. Short Stature in Growth Hormone Deficiency

The GH–IGF-1 axis is a pivotal regulator for normal musculoskeletal development in children, as its integrity is essential for both fetal and postnatal growth. Consequently, genetic alterations affecting various components of this axis can break the physiological continuum of the growth process, giving rise to disorders ranging from mild to severe, depending on the degree of GH or IGF-1 abnormality. In most cases, these mutations result in a proportionate short stature phenotype, characterized by harmonious skeletal proportions and the absence of major organ anomalies [17]. However, in other situations—such as when GHD is part of a syndromic disorder or if associated with combined pituitary hormone deficiency (CPHD)—additional abnormalities may be present, involving the visual system, such as anophthalmia, or the central nervous system, such as structural brain malformations including septo-optic dysplasia and posterior pituitary ectopia [12]. Mutations in genes correlated with GHD may occur at different levels, involving a host of pathways and finally causing isolated or combined deficiency of GH, GH insensitivity, and IGF-I insensitivity, respectively.

## 4. Growth Hormone Deficiency

GHD may occur isolated or in association with the deficiency of other hormones of the hypothalamic–pituitary axis, as part of a broader condition known as “hypopituitarism”. Mutations in genes involved in the pituitary development can lead to congenital hypopituitarism: this condition may present as either CPHD, where two or more anterior pituitary hormones are affected, or as isolated hormone deficiencies, with IGHD being the most common [12,16,18]. The genes implicated in these disorders can be broadly categorized into three functional groups:

– Genes involved in GH synthesis and secretion: *GH1* and *GHRH* (see Table 1).
– Genes involved in pituitary organogenesis: some examples include *GLI2*, *HESX1*, *LHX3*, *LHX4*, *SOX2*, *SOX3*, *OTX2*, etc., (see Table 2).
– Genes regulating pituitary cell lineage differentiation (particularly somatotrophs): *POU1F1*, *PROP1*, *IGSF1*, and *ZBTB20* (see again Table 2).

Short stature associated with growth hormone deficiency can also result from mutations in genes not traditionally linked to growth regulation. Recent findings identify missense mutations in KCNQ1, a gene previously known for its role in cardiac arrhythmias, as a novel genetic cause of growth hormone deficiency [19]. The expression of KCNQ1 in hypothalamic growth hormone-releasing hormone neurons and pituitary somatotropes suggests its involvement in the neuroendocrine control of growth [20].

### 4.1. Isolated Growth Hormone Deficiency

A major category of genetic causes of IGHD includes mutations that affect either production or activity (signal transduction) of the GH, primarily involving the *GH1* and *GHRHR* genes. These mutations are estimated to account for approximately 3 to 30% of all IGHD cases, with *GH1* variants being more frequent than *GHRHR* ones [15]. The *GH1* gene is located on chromosome 17q22.24, and its mutations are responsible for several distinct forms of IGHD, including IGHD type IA, IGHD type IB (also associated with *GHRHR* mutations), IGHD type II, and Kowarski syndrome (also known as “bioinactive GH syndrome”). IGHD IA is inherited in an autosomal recessive manner and is most commonly caused by homozygous deletions or nonsense mutations in *GH1*, leading to the complete absence of GH in the serum, followed by severe early growth failure that becomes evident within the first 6 months of life. Although an initial response to GH therapy may be observed, many patients develop anti-GH antibodies [21], which consistently reduce treatment efficacy. In such cases, IGF-1 therapy may be eventually considered. IGHD type IB can result from mutations in either *GH1* or *GHRHR*, both inherited in an autosomal recessive pattern; these patients typically exhibit low GH levels in the stimulation tests, though phenotypes are milder than type IA with no development of anti-GH antibodies after GH therapy. *GH1* mutations in patients with IGHD type IB may include splice site, missense, or nonsense variants [12,22]. *GHRHR* abnormalities are heterogeneous, and may be characterized by missense, nonsense, splice site mutations, microdeletions, or promoter alterations [17]. The clinical features may include low IGF-1 and IGFBP-3 levels with anterior pituitary hypoplasia [23]. IGHD type II, the most common genetic subtype, is caused by autosomal-dominant mutations in *GH1*, typically splice site or missense mutations [22]: the most common mechanism involved is an aberrant splicing of exon 3, resulting in a GH isoform with a dominant-negative effect: specifically, the isoforms have a faulty structure which interferes with normal GH secretion by trapping it inside the endoplasmic reticulum through the formation of aggregates, with the consequence that neither normal nor pathological GH is properly secreted [24]. IGHD type II often presents with significant phenotypic variability, affecting both stature and pituitary morphology. Additional anterior pituitary hormone deficiencies (e.g., ACTH, TSH, FSH, LH, and prolactin) can also frequently be observed [25]. Kowarski syndrome results from autosomal-dominant mutations in *GH1*, that produce biologically inactive GH with reduced affinity for the GH receptors (GHRs) and GH-binding proteins (GHBPs). Despite elevated serum levels of GH, patients show low levels of IGF-1 and IGFBP-3 [22]. However, they typically respond well to recombinant GH therapy, often achieving remarkable linear growth [17]. Recent studies suggest that mutations in the *GHSR* gene (encoding the ghrelin receptor) may contribute to partial IGHD. These variants can be inherited in either a dominant or recessive manner, and the clinical presentation may vary, often including short stature associated with constitutional delay of growth and puberty [26,27]. Serum GH and IGF-1 concentrations can be variable. Inoue et al. showed how mutations of *GHSR1A* can contribute to the etiology of short stature in a Japanese population [28], but further research is needed to better clarify the role of such mutations and confirm these preliminary data. IGHD type III is a rare disorder caused by mutations in *BTK* and *SOX3* genes, which follows an X-linked inheritance pattern. A *BTK* mutation resulting in exon skipping was identified in one patient with both GH deficiency and X-linked agammaglobulinemia [29].

### 4.2. Genes Involved in Pituitary Development

GHD may be also associated with mutations in genes involved in pituitary ontogenesis and somatotroph differentiation: when hormone deficiencies are multiple, the underlying cause should be investigated as both organic pathology (such as oncological, post-traumatic, or infectious diseases of the central nervous system) and a genetic defect might be present, particularly in cases with early-onset symptoms during the neonatal period [30,31]. In this context, there are many genes involved: *LHX3* and *LHX4*, for instance, are essential transcription factors involved in early pituitary development, as they encode LIM-homeodomain proteins expressed in Rathke’s pouch; these proteins are critical for proper formation of the anterior pituitary gland. *LHX3* mutations, inherited in a recessive manner, are associated with CPHD and typically give a triad of features: combined pituitary hormone deficiency, sensorineural hearing loss, and cervical spine anomalies (including short neck with limited rotation, cervical spina bifida occulta, and rigid cervical spine) [31]. Brain magnetic resonance imaging (MRI) findings may reveal a normal or hypoplastic and even enlarged anterior pituitary [12]. *LHX4* mutations, usually inherited in a dominant pattern, cause a variable spectrum of pituitary dysfunction ranging from IGHD to CPHD [32]. Unlike *LHX3*, *LHX4* variants are not associated with sensorineural deafness, but may be associated with cerebellar abnormalities, including Arnold–Chiari malformation [33]. Brain MRI findings may also include anterior pituitary hypoplasia and ectopic posterior pituitary [34]. *HESX1*, instead, is a transcriptional repressor that plays a role in early forebrain and pituitary development. Both autosomal-dominant and -recessive mutations in *HESX1* have been associated with a spectrum of hormonal deficiencies, ranging from IGHD to CPHD with panhypopituitarism [35]. Clinical features may include developmental delay, while brain MRI may show anterior pituitary hypoplasia, ectopic posterior pituitary, or septo-optic dysplasia [34,36]. Among the genes included in pituitary ontogenesis, there is also *OTX2*, a homeobox gene involved in the development of the hypothalamic–pituitary axis, eyes, and central nervous system. Mutations in *OTX2*, which may follow either dominant or recessive inheritance, have been associated with IGHD and CPHD. Clinical manifestations may include microcephaly, bilateral anophthalmia or microphthalmia, developmental delay, and cleft palate [37]. Brain MRI findings often show anterior pituitary hypoplasia or ectopic posterior pituitary [12]. Conversely, *SOX3* is a gene that contributes to the development of Rathke’s pouch and normal function of the hypothalamic–pituitary axis [38]. *SOX3* variants follow an X-linked inheritance pattern, and are associated with a clinical spectrum ranging from IGHD to CPHD. Affected individuals may also present with cognitive impairment, mostly intellectual disability and learning difficulty [39]. In addition to pituitary abnormalities, brain MRI may reveal structural anomalies such as corpus callosum dysgenesis. Also *SOX2*, similarly to *SOX3*, plays a role in Rathke’s pouch formation and anterior pituitary development [38]. *SOX2* mutations, predominantly related to autosomal-dominant inheritance, though with rare recessively inherited cases, are associated with a broad spectrum of clinical features, including optic nerve hypoplasia, micropenis, sensorineural hearing loss, gastrointestinal malformations, and central nervous system abnormalities [12,19,36]. These patients usually present CPHD [10]. Talking of *GLI2*, it can be considered a key effector of the sonic hedgehog (SHH) signaling pathway; heterozygous mutations in *GLI2* are associated with a broad spectrum of phenotypes ranging from holoprosencephaly to CPHD [40,41]. Additional clinical features may include cleft lip and/or palate, anophthalmia, post-axial polydactyly, but also imperforate anus, laryngeal cleft, renal agenesis, and anterior pituitary hypoplasia [19,42]. *GLI3* is another gene which participates in the SHH signaling, being implicated in developmental processes. Heterozygous *GLI3* mutations can lead to CPHD and are classically associated with the Pallister–Hall syndrome; further clinical findings may include hypothalamic hamartoblastoma and post-axial polydactyly, with brain MRI showing hypoplastic anterior pituitary [43]. With regard to midline development, *FGF8* and *FGFR1* genes can be considered essential for the pathogenesis of Kallmann syndrome and pituitary dysgenesis [44]. More specifically, homozygous mutations in *FGF8* have been linked to septo-optic dysplasia, holoprosencephaly, Moebius syndrome, and also maxillary hypoplasia, microcephaly, and spastic diplegia [36,42], while *FGFR1* mutations inherited in a dominant manner have been associated with anterior pituitary hypoplasia, agenesis of the corpus callosum, and ocular anomalies [42,45]. *PROKR2*, on the other hand, is a gene involved in the migration of gonadotropin-releasing hormone (GnRH) neurons, known to contribute to Kallmann syndrome. However, heterozygous mutations in *PROKR2* have also been implicated in pituitary developmental anomalies and CPHD, with variable presentations of hypopituitarism [46]. The main associated clinical features include clubfeet, syringomyelia, microcephaly, and epilepsy. Brain MRI findings can reveal anterior pituitary hypoplasia [42,44]. Furthermore, there is also the gene *CDON*, encoding a co-receptor in the SHH signaling pathway, which cooperates in midline and pituitary development and whose heterozygous mutations are associated with CPHD, commonly involving deficiencies of GH, ACTH, and TSH; in these cases, a brain MRI might reveal an absent pituitary stalk with posterior pituitary ectopia [45]. *ARNT2* is a gene encoding a transcription factor involved in regulation of the hypothalamic–pituitary axis, and its homozygous mutations can result in CPHD, frequently accompanied by antidiuretic hormone deficiency; brain MRI findings include ectopic or absent posterior pituitary, thin pituitary stalk, delayed myelination, and hypoplasia of the corpus callosum. Further clinical manifestations include microcephaly, prominent forehead, deep-set eyes, retrognathia, hip dysplasia, hydronephrosis, vesicoureteral reflux, and neurogenic bladder [12,46]. The set of involved genes may also include *ROBO1*, which has a seminal role in steering pituitary stalk and hypothalamus development; rare heterozygous mutations may cause CPHD with central hypothyroidism, but some patients may also display ocular anomalies such as strabismus and ptosis [47]. *TBC1D32* is a ciliary gene involved in SHH signaling and its mutations, transmitted via the recessive pattern, can result in a clinical spectrum ranging from IGHD to panhypopituitarism with syndromic features, including facial dysmorphism, retinal dystrophy, and developmental delay [48]. Another gene of interest is HMGA2, which plays a role in the regulation of stem cell proliferation. Heterozygous variants in HMGA2 have been described as a monogenic cause of Silver–Russell syndrome (SRS), a condition typically characterized by severe growth failure. While most individuals with HMGA2-related SRS do not present with structural pituitary abnormalities or overt growth hormone deficiency, a minority of cases have been reported with central GHD and abnormal brain MRI findings, including ectopic posterior pituitary. Therefore, although HMGA2 is not a classical pituitary gene, its involvement in growth regulation and occasional association with GHD warrant further investigation [23,49]. There are also other genes associated with ciliopathies, such as *ALMS1* and *IFT172*, which contribute to syndromic forms of pituitary dysfunction if biallelic mutations are present. *ALMS1* variants, for instance, cause Alström syndrome, with approximately 50% of cases showing GHD [50,51]; *IFT172* variants, in contrast, can also lead to GHD with other additional features such as retinopathy, metaphyseal dysplasia, renal impairment, and hypertension. A brain MRI may typically reveal pituitary abnormalities [49]. Mutations that lead to CPHD can also involve *PITX2*, a gene that encodes a transcription factor essential for eye, tooth, and pituitary development: in this case, the multiple hormone deficiency associated is present in the context of Rieger syndrome. Further clinical features may include ocular defects, dental anomalies, and pituitary dysfunction [52]. Furthermore, *EIF2S3* is also an X-linked gene that can be implicated in rare cases of CPHD, associated with glucose metabolism disorders such as hyperinsulinemic hypoglycemia and post-prandial hypoglycemia. Additional features may include intellectual disability and microcephaly [53]. Another important gene to consider is *MAGEL2*, which plays a critical role in regulating the hypothalamic–pituitary axis and controlling GH secretion, its heterozygous mutations on the paternal allele are associated with the Prader–Willi syndrome spectrum, which includes GHD as part of a more complex broader phenotype. Other common features may encompass neonatal hypotonia, obesity, developmental delay, joint contractures, and dysmorphic features, while athrogryposis may also be observed in a minority of cases; brain MRI, in this circumstance, might disclose a small posterior pituitary with optic nerve hypoplasia [54]. Moreover, it is important to mention *L1CAM*, a gene located on the X chromosome, that may be rarely associated with brain malformations in the context of GHD or syndromic CPHD: general clinical features include hypopituitarism, hydrocephalus, ventricular septal defect, developmental delay, astigmatism, arthrogryposis, and scoliosis [55]. *GRP161* encodes a protein that acts as a negative regulator of the SHH signaling pathway, and in rare cases its homozygous mutations have been associated with CPHD. Additional features of these mutations include congenital ptosis, alopecia, syndactyly of the second and third fingers, and nail hypoplasia; brain MRI findings, moreover, typically include a small anterior pituitary and ectopic posterior pituitary [56]. Proceeding further, there is *RNPC3*, that encodes a core component of the minor spliceosome that is essential for splicing U12-type introns: biallelic variants in this gene have been associated with CPHD, also being accompanied by GHD, central congenital hypothyroidism, delayed puberty, congenital cataracts, and developmental delay [57]. *LAMB2* encodes an extracellular matrix protein crucial for pituitary morphogenesis, and biallelic *LAMB2* mutations can lead to GHD, developmental delay, focal segmental glomerulosclerosis, and congenital nephrotic syndrome, while a brain MRI might reveal anterior pituitary hypoplasia and optic nerve hypoplasia [58]. In addition, there is *TCF7L1*, a gene that encodes a key effector of the Wnt/β-catenin signaling pathway, contributing to early forebrain and pituitary development: its heterozygous missense mutations, inherited in a dominant fashion with variable penetrance, have been identified in patients with isolated GHD or reduced IGF-1 levels [57,58]. Mutations in NR0B1 (DAX1) are well established as the cause of X-linked congenital adrenal hypoplasia, primarily characterized by adrenal insufficiency and hypogonadotropic hypogonadism. Recent studies [59] have identified growth hormone deficiency as a novel clinical feature associated with NR0B1 mutations, expanding the phenotypic spectrum of this disorder. This finding suggests that DAX1 plays a broader role in hypothalamic–pituitary axis regulation beyond adrenal and gonadal function, contributing to impaired growth in affected patients. Finally, the gene *RAX*, encoding a transcription factor crucial for the development of eyes, forebrain and pituitary, can display recessively inherited mutations linked to congenital hypopituitarism. Also anophthalmia, bilateral cleft lip and palate, or complete absence of the pituitary gland can be found as further associated symptoms [60].

### 4.3. Genes Mainly Involved in Pituitary Somatotroph Differentiation

Among the genes contributing to pituitary development, some play a main role in somatotroph differentiation. *PROP1*, for instance, encodes a transcription factor with a double activity: activating the expression of *POU1F1* and repressing the expression of *HESX1*. *PROP1* mutations represent the most common genetic cause of CPHD, and clinical presentation is mutation-dependent, starting as either IGHD or CPHD, typically involving deficiency in GH, prolactin, TSH, and gonadotropins LH and FSH, with ACTH deficiency occurring less frequently. Gonadotropin deficiency is highly variable and may present with delayed puberty, micropenis or cryptorchidism [12,61]. *POU1F1* (also known as *PIT1*) is activated by *PROP1* and encodes a transcription factor that drives somatotroph differentiation by directly regulating *GH1* expression; mutations in *POU1F1* lead to GHD combined with a deficiency in TSH and prolactin, with a clinical onset that may vary from birth to adolescence [62]. The phenotype is highly variable and mutation-dependent, ranging from isolated IGHD to CPHD [63,64]. *POU1F1* is considered a master regulator of GH-producing cells; furthermore, it also regulates the expression of *GHRHR* and *GH1*. A recent study demonstrated that high-level *GH1* expression requires *POU1F1* binding to two specific loci in the proximal promoter region of *GH1*, referred to as LCR1 and LCR2. Therefore, mutations within these loci could steer IGHD [20]. Another relevant role is played by *IGSF1*, located on the X chromosome, influencing both thyrotroph and somatotroph function. In particular, IGSF1 deficiency, caused by loss-of-function *IGSF1* mutations, is characterized by central hypothyroidism and macroorchidism [65,66,67,68], with a few cases showing GHD [65]. The pattern of transmission is X-linked, and additional clinical features can include delayed puberty and frontoparietal hygroma or in some cases lesions of the pituitary stalk [12,68]. Finally, there is *ZBTB20*, that encodes a transcription factor which modulates *GH1* expression in the anterior pituitary; autosomal-dominant mutations with variable penetrance or dysregulation of *ZBTB20* can impair *GH1* transcription, resulting in IGHD. These patients typically present a proportionate short stature, but delayed growth velocity [67,68].

## 5. Growth Hormone Insensitivity

The secretion of GH is primarily regulated by GH releasing hormone (GHRH), which originates from the hypothalamus and stimulates both the secretion of stored GH from the anterior pituitary and the transcription of the *GH1* gene, which encodes the GH protein [69]. Once secreted, GH circulates in the bloodstream, where a portion binds to GHBP, prolonging the hormone’s half-life and modulating its bioavailability to target tissues. GH exerts its effects by binding to pre-dimerized GH receptors (GHRs) on the cell surface: this interaction induces conformational changes that activate Janus kinase 2 (JAK2), a tyrosine kinase that allows GH signal transduction [70]. JAK2 phosphorylates signal transducers and activators of transcription (STAT) proteins, mainly STAT5A and STAT5B, which then translocate to the nucleus to regulate the expression of target genes [71]. Beyond the JAK/STAT pathway, GHR activation also triggers PI3K/AKT, MAPK/ERK, and calcium signaling pathways, influencing cell proliferation, metabolic homeostasis, and growth plate development as well as muscle and adipose tissue metabolism [72]. One of GH’s major downstream effects is the stimulation of IGF-1 synthesis, primarily within the liver and other peripheral tissues. IGF-1 mediates many of the growth-promoting effects of GH. In circulation, IGF-1 forms ternary complexes with IGF-binding proteins (IGFBPs) and acid-labile subunits (ALS), which stabilize IGF-1 and extend its half-life. IGF-1 binds to the IGF-1 receptor (IGF-1R) on target cells, activating multiple intracellular signaling cascades that promote cell growth, survival, and metabolic reactions [73]. While insensitivity to GH (GHI) is characterized by low IGF-1 levels, normal or elevated GH levels, and lack of IGF-1 response to GH treatment, IGF-1 resistance is characterized by elevated IGF-1 levels with normal or high GH levels [74]. Genetic defects involved in GHI include two main categories: Laron syndrome, associated with defects within *GHR* gene, and alterations that cause defects in the GH intracellular signaling pathway, such as mutations of *STAT5B*, *IKBKB*, *STAT3*, *IL2RG* and *PIK3R1*.

### 5.1. Defects in the Growth Hormone Receptor Gene (Laron Syndrome)

Laron syndrome, first described in 1966, was the first recognized cause of GHI [75], most commonly caused by homozygous mutations in the *GHR* gene, which encodes the GHR [76]. However, autosomal-dominant inheritance patterns have also been reported in the medical literature [77,78]. Mutations associated with Laron syndrome are diverse, including splice-site, nonsense and missense variants [79]. These can affect different domains of the GHR: the extracellular, transmembrane, or intracellular regions. GHBP levels may serve as diagnostic clues, as they are typically absent in mutations affecting the extracellular domain, but are present in cases related to intracellular or transmembrane mutations [12]. Talking about the biochemical profile, it typically includes normal or elevated basal GH levels, an exaggerated GH response to stimulation tests and low-serum levels of IGF-1, IGFBP-3 and ALS. The clinical phenotype of Laron syndrome, instead, shares similarities with severe GHD, including midface hypoplasia, frontal bossing and proportionate short stature [22,79]. Diagnosis relies on a combination of clinical, biochemical, and genetic data. The IGF-1 generation test, an endocrinological test used to evaluate body’s ability to produce IGF-1 after GH administration, may support the diagnosis, although it has a moderate-to-high sensitivity and should not be used as a single test [80].

### 5.2. Defects in Intracellular Growth Hormone Signaling Pathway

The clinical phenotype of GHI with specific immunity dysfunction can be caused by various genes: *STAT5B*, for instance, encodes for a critical transcription factor within the intracellular GH signaling cascade, which plays a contributive role in IGF-1 production. While defects in other STAT proteins are associated with immunodeficiencies, only *STAT5B* mutations result in GHI due to impaired IGF-1 biosynthesis. Both homozygous recessive and dominant mutations in *STAT5B* have been associated with IGF-1 deficiency, postnatal growth failure, immune dysregulation, and pulmonary fibrosis [81,82]. Delayed bone age and delayed puberty are common [81,82,83], and probably related to chronic illness. The biochemical profile typically includes elevated GH levels and markedly low levels of IGF-1, IGFBP-3, and ALS, with a growth pattern resembling Laron syndrome [12]. *STAT3* is involved in many immune system processes, and heterozygous gain-of-function *STAT3* mutations, inherited in a dominant manner, have been associated with early-onset multisystem autoimmune diseases, including type 1 diabetes [84]. These mutations can impair the phosphorylation of STAT1 and STAT5 proteins and T-cell function, resulting in a phenotype that includes short stature alongside immune dysregulation [85]. Moreover, there is the gene *IKBKB*, which encodes the IκB kinase β (IKKβ), a member of the NF-κB signaling pathway which has the aim of regulating gene expression by controlling the activation of NF-κB transcription [86]. *IKBKB* mutations, specifically heterozygous variants, have been reported in patients with growth retardation, partial GH and IGF-1 insensitivity, and immunologic abnormalities, suggesting a broader impact of NF-κB signaling on growth and endocrine regulation [87]. It is important to mention also *IL2RG*, that encodes the common γ-chain (γc) of interleukin receptors, essential for cytokine signaling: mutations in this gene are the primary cause of X-linked severe combined immunodeficiency [88]. In these patients, growth failure is linked to impaired STAT5B protein phosphorylation and nuclear translocation, leading to severe IGF-1 resistance [87,88]; they also show minimal or no response to GH therapy, both in terms of linear growth and serum IGF-1 elevation [88]. Another recently described genetic cause of growth hormone insensitivity is linked to biallelic pathogenic variants in *QSOX2*, a gene encoding a nuclear membrane protein with disulfide isomerase and oxidoreductase activity. A recent study [89,90] identified five patients from three unrelated families presenting with syndromic short stature, gastrointestinal dysmotility, immune dysfunction, and atopic eczema, all harboring recessive *QSOX2* mutations. Functional studies revealed that *QSOX2* deficiency impairs the nuclear translocation of phosphorylated STAT5B in response to growth hormone, despite normal or even enhanced GH-induced STAT5B phosphorylation. Furthermore, patient-derived fibroblasts exhibited GH-induced mitochondrial dysfunction, indicating that *QSOX2* plays a dual role in regulating GH signaling and mitochondrial dynamics [89].

Finally, there is *PIK3R1*, a gene involved in the PI3K/AKT/mTOR pathway, which promotes cell proliferation and survival [91]; heterozygous mutations in *PIK3R1* cause the so-called SHORT syndrome, characterized by short stature (S), hyperextensibility of joints and/or inguinal hernia (H), ocular depression (O), Rieger abnormality (R) and teething delay (T) [92]. In addition to growth failure, a host of immune abnormalities can be commonly observed in these patients [13]. See Table 3 for more information.

## 6. Insulin-like Growth Factor I Resistance

IGF-1 circulates in the blood stream bound to IGFBP3 (or IGFBP5) and to ALS (acid labile subunit), creating a tertiary complex that lengthens the IGF-1 half-life [73]. Studies conducted in families with *IGFALS* mutations showed that patients with both homozygous and heterozygous mutations had lower levels of IGF1 and IGFBP3, with final height and head circumference being smaller compared to healthy controls [93]. The mechanisms mainly involved are synthesis of insulin-like growth factors; isolated IGF deficiency (IGF1, IGF2), transport/bioavailability of IGFs; ternary complex defect (due to mutations in *IGFALS*, *PAPPA2* and *STC2*); and IGF-1 sensitivity (due to mutations in *IGF1R*).

### 6.1. Defective Synthesis of Insulin-like Growth Factor-1 and of Insulin-like Growth Factor-2

Homozygous mutations or deletions in *IGF1* are rare, but can result in GHI with clinical features including birth of newborns small for gestational age and postnatal growth impairment, microcephaly with developmental delay, and hearing loss [94,95,96]. Among the biochemical findings, instead, we find elevated baseline and peak GH levels, but very low serum IGF-1 concentrations and normal IGFBP-3 and ALS levels [42,97]. Treatment options for *IGF1* mutations include human recombinant IGF-1 therapy. However, for patients with *IGF1* deletions, therapy may be less effective due to a higher risk of developing IGF-1 antibodies [12]. In cases of heterozygous mutations, Fuqua et al. showed that growth failure tends to be less severe [98]. Begemann et al. investigated a multi-generational family with individuals exhibiting growth restriction and dysmorphic features, finding that the phenotype was linked to the paternal inheritance of a pathogenic variant of *IGF2*; these findings suggested that IGF2 is crucial for both pre- and postnatal growth [99]. Additionally, IGF2 is also implicated in growth retardation associated with Silver–Russell syndrome, a genetically heterogeneous condition which is largely characterized by IGF-1 insensitivity with hypomethylation of the imprinting control region 1 at the IGF2/H19 locus on 11p15 [100].

### 6.2. Ternary Complex Defect

Children with ALS deficiency, inherited in a recessive pattern, typically present with mildly stunted growth, delayed puberty, hyperinsulinism, and, in some cases, osteopenia. Biochemical findings include very low serum levels of IGF-I, IGFBP-3 and ALS, with variable GH levels [101]. Heterozygosity for *IGFALS* variants can lead to short stature with lower final height and head circumference if compared to age-matched controls [102]. The *PAPPA2* gene is involved in regulating IGF-1 bioavailability, which codifies a protein that cleaves IGFBP-3 and IGFBP-5, thus increasing the activity of IGF-1 [103]; loss of function mutations in *PAPPA2* result in increased IGF-1 bound to the ternary complex, leading to decreased levels of free IGF-1 and subsequent short stature. Clinical manifestations can include skeletal abnormalities, microcephaly, elevated serum levels of GH, IGF-1, IGFBP-3 and ALS [104]. The *STC2* is a gene that encodes stanniocalcin 2, a potent inhibitor of the PAPP-A and PAPP-A2 proteins, which mostly regulates IGF-1 bioactivity. With regard to *STC2* mutations, even if there are no documented cases of pathogenic *STC2* variants in the medical literature, experimental evidence in mice suggests that loss-of-function *STC2* mutations might lead to tall stature, while gain-of-function mutations to growth impairment. Overexpression of *STC2* in mice leads to decreased growth, supporting the hypothesis that *STC2* overactivity results in stunted growth [105].

### 6.3. Insulin-like Growth Factor-1 Resistance

Mutations in the gene *IGFR1*, coding for IGFR are typically heterozygous, as the complete absence of IGFR1 might be lethal [87]. Such variants can be transmitted with both recessive and dominant inheritance patterns: homozygous and compound heterozygous mutations tend to be more severe [106,107]. The impairment of IGFR action might be associated with different mechanisms, including haploinsufficiency of the receptor, lower binding affinity, interference in signaling, reduced biosynthesis, and disruption of the tyrosine kinase activity. The clinical phenotype often includes pre- and postnatal growth failure with microcephaly [108]. The impact of *IGFR1* on intrauterine growth is more significant if maternally inherited, probably because it leads to reduced placental development [109]. Growth impairment can also be caused by either mutations downstream of the *IGFR* or decreased microRNA regulation of the IGF-1 pathway [110]. Biochemical findings include normal or higher levels of IGF-1 in the serum, while treatment with GH does not seem effective [108]. More information is reported in Table 4.

## 7. Discussion

The most recent progress in the evaluation protocols of children with short stature has led to the identification of an increasing number of genetic variants associated with abnormalities in the GH–IGF-1 axis, all variably contributing to linear growth. It is hypothesized that many cases currently classified as “idiopathic’” short stature or as IGHD may, in fact, harbor underlying genetic mutations predisposing to poor or insufficient growth [111,112]. At present, genetic testing is not routinely recommended as a first-line investigation tool for patients with short stature [113], but is indeed reserved for children presenting with specific features such as disproportionate short stature, height significantly below mid-parental target range, or distinctive dysmorphic features [114]. Interestingly, many of the mutations discussed in this review are associated exclusively with short stature in the absence of additional phenotypic features. As a result, many of these cases may go undiagnosed for a very long period of time, and may be unknowingly transmitted to subsequent generations [111]. It is also established that, among various genetic investigations conducted in children with short stature, certain mutations are examined with priority due to their relatively higher prevalence in the general population [113]. A prime example is the *SHOX* gene [115,116,117], which represents a common genetic cause of short stature with normal GH secretion and either normal or variable responses to GH stimulation tests [117]. Its associated phenotypes can range in severity depending on the specific mutation involved. Currently, according to the current guidelines, patients with *SHOX* haploinsufficiency are eligible for GH therapy and often demonstrate treatment responses comparable to those observed in patients with classic idiopathic GHD [118,119].

Among the genetic causes of short stature, the most frequently reported ones are associated with multiple defects in the hypothalamic–pituitary function [111,112,113,114]. These conditions may acutely occur during the neonatal period due to deficiencies in hormones other than GH [120]. This category includes mutations involved in CPHD, that may be associated with autosomal-dominant, autosomal-recessive and X-linked patterns of inheritance, as previously discussed, being also associated with either syndromic or non-syndromic pictures. However, their diagnosis can be challenging even in the most suggestive cases, as genotype–phenotype correlations may be non-linear. It is not uncommon, for instance, in patients with *HESX1* mutations [28], despite their autosomal-dominant inheritance, to be completely asymptomatic or to exhibit very mild clinical manifestations [28,29,30]. In many cases, when gene mutations are closely associated with IGHD and CPHD, GH therapy seems effective in the treatment of short stature [12]. However, among genetic causes of short stature, it is also important to consider those conditions that, despite not involving a direct defect on GH secretion and activity, are nonetheless associated with impaired growth and may benefit from GH therapy [113]. A classic example is given by girls with Turner syndrome, characterized by a karyotype containing one X chromosome and complete or partial absence of the second X chromosome, in whom short stature and normal response to GH stimulation tests are very commonly observed, representing one of the main indications approved for GH treatment [121]. In addition, there are numerous other genes that are implicated in growth impairment, but have not yet been clearly associated with various short stature phenotypes [111]. As a matter of fact, chronic inflammation in children can be associated with growth retardation, and growth delay as well as pubertal growth spurt delay are commonly encountered in patients with autoinflammatory diseases, a group of inherited disorders of the innate immune system caused by mutations within protean genes involved in either regulation or activation of the inflammatory response [122]. Novel advances in our understanding of inflammatory processes should ultimately lead to more effective and specific means of interventions to stimulate the functional activity of different target cells at the growth plates or cells actively impacted by the GH-IGF1 axis. Some clinical scores have been created to quantify damage in both adult and pediatric patients with autoinflammatory diseases impacting on the skeleton health and to compare disease outcomes in clinical studies [123,124]. In particular, somatic growth may be altered in patients with cryopyrin-associated periodic syndrome, a rare dominantly inherited autoinflammatory disease prevalently driven by uncontrolled production of interleukin-1 and caused by missense mutations in the *NLRP3* gene, chiefly involved in the processing of interleukin-1 and pyroptosis [125]. Cryopyrin-associated periodic syndrome is associated with growth deficiency starting as intrauterine growth retardation and aberrant endochondral bone growth [126]. These children have an interleukin-1-based inflammation affecting various systems, such as the central nervous system and bones, which can lead to permanently severe growth disturbance, sometimes even related to GHD, even showing a substantial response to GH replacement [127]. Conversely, data related to the long-term benefits of a treatment with interleukin-1 antagonists on the somatic growth of children with cryopyrin-associated periodic syndrome are so far not available [128]. Although the genetic causes of intrauterine growth restriction are not clearly defined, some researchers have shown both effectiveness and safety of GH treatment in small-for-gestational-age patients regardless of the genetic results in genes regulating growth cartilage development at the whole-exome sequencing study [129].

In conclusion, the present review has several limitations: it is a narrative review, and it is plausible that relevant articles might not have been identified and retrieved during the screening process. Furthermore, we did not examine other etiologies of short stature unrelated to GH pathway impairment, such as defects affecting cartilage extracellular matrix, paracrine factors in the growth plate, or genetic defects directly affecting cellular or intracellular processes. Nonetheless, this study of ours offers a comprehensive analysis that summarizes the most recent evidence dealing with the genetically based causes of short stature in children displaying GH deficiency and provides an updated framework that could be helpful in finding new diagnostic and therapeutic strategies for children affected by disruption of the GH-IGF1 axis.

## 8. Conclusions

This scoping review highlights the most relevant genetic diagnoses of children presenting with short stature related to GHD. The recent advancement in understanding the genetic background of GHD has significantly improved both diagnostic precision and clinical management of short stature in pediatric patients. Ongoing research into novel genetic variants and genotype–phenotype correlations will further deepen our in-depth knowledge of the GH–IGF-1 axis and point out its exact multi-tasking contribution to linear pediatric growth.

## Figures and Tables

**Table 1:** Genes involved in isolated growth hormone deficiency.

Gene	Syndrome/IGHD Type	Inheritance	Clinical and Labwork Features
*GH1*	IGHD IA	AR	Severe GHD starting in infancy, undetectable GH, anti-GH antibodies, poor GH therapy response, use of IGF-1 therapy
*GH1*	IGHD IB	AR	Low GH on stimulation test, milder phenotype, no anti-GH antibodies, low IGF-1/IGFBP-3, pituitary hypoplasia
*GH1*	IGHD II	AD	Variable phenotype, dominant-negative exon 3 splicing effect, in some cases also combined pituitary hormone deficiencies can be found
*GH1*	Kowarski syndrome	AD	Bioinactive GH, high GH but low IGF-1/IGFBP-3, good response to GH therapy
*GHRHR*	IGHD IB	AR	Similar to GH1 IB: low GH, low IGF-1/IGFBP-3, pituitary hypoplasia, no anti-GH antibodies
*GHSR*	Partial IGHD	AD or AR	Short stature, constitutional growth/puberty delay, variable GH/IGF-1 levels
*BTK*	IGHD III	X-linked recessive	GH deficiency with X-linked agammaglobulinemia
*SOX3*	IGHD III	X-linked recessive	IGHD with cognitive impairment, corpus callosum anomalies, pituitary abnormalities

AD: autosomal dominant; AR: autosomal recessive; GH: growth hormone; GHD: growth hormone deficiency; IGHD: isolated growth hormone deficiency.

**Table 2:** Genes involved in pituitary development.

Gene	Inheritance	Phenotype	Clinical and Labwork Features
*LHX3*	AR	CPHD	Sensorineural deafness, cervical spine anomalies, short neck, normal/enlarged or hypoplastic anterior pituitary
*LHX4*	AD	IGHD → CPHD	Cerebellar anomalies (e.g., Arnold–Chiari anomaly), anterior pituitary hypoplasia, ectopic posterior pituitary
*HESX1*	AD or AR	IGHD → CPHD	Developmental delay, anterior pituitary hypoplasia, ectopic posterior pituitary, septo-optic dysplasia
*OTX2*	AD or AR	IGHD/CPHD	Microcephaly, microphthalmia or anophthalmia, developmental delay, cleft palate, anterior pituitary hypoplasia
*SOX3*	X-linked	IGHD → CPHD	Cognitive impairment, corpus callosum dysgenesis, pituitary abnormalities
*SOX2*	AD (rarely AR)	CPHD	Optic nerve hypoplasia, micropenis, hearing loss, GI and CNS malformations
*GLI2*	AD	CPHD	Holoprosencephaly spectrum, cleft lip/palate, polydactyly, renal/anal anomalies, anterior pituitary hypoplasia
*GLI3*	AD	CPHD	Pallister–Hall syndrome, hypothalamic hamartoma, postaxial polydactyly, hypoplastic anterior pituitary
*FGF8*	AR	CPHD	Septo-optic dysplasia, Moebius syndrome, microcephaly, midline defects
*FGFR1*	AD	CPHD	Anterior pituitary hypoplasia, corpus callosum agenesis, ocular anomalies
*PROKR2*	AD	CPHD	Clubfeet, syringomyelia, epilepsy, microcephaly, anterior pituitary hypoplasia
*CDON*	AD	CPHD	GH, ACTH, TSH deficiencies; absent stalk, ectopic posterior pituitary
*ARNT2*	AR	CPHD	ADH deficiency, delayed myelination, microcephaly, urogenital anomalies
*ROBO1*	AD	CPHD	Central hypothyroidism, strabismus, ptosis
*TBC1D32*	AR	IGHD → CPHD	Facial dysmorphism, retinal dystrophy, developmental delay
*HMGA2*	AD	CPHD	Severe GHD, ectopic posterior pituitary
*ALMS1*	AR	GHD (CPHD syndromic)	Alström syndrome, GHD in 50% of the cases
*IFT172*	AR	GHD (CPHD syndromic)	Retinopathy, metaphyseal dysplasia, renal issues
*PITX2*	AD	CPHD (Rieger syndrome)	Ocular and dental anomalies
*EIF2S3*	X-linked recessive	CPHD	Glucose metabolism disorders, intellectual disability, microcephaly
*MAGEL2*	Paternal allele mutation	CPHD (syndromic)	Prader–Willi features: hypotonia, obesity, developmental delay, joint contractures
*L1CAM*	X-linked recessive	CPHD (syndromic)	Hydrocephalus, VSD, scoliosis, developmental delay
*GRP161*	AR	CPHD	Ptosis, alopecia, syndactyly, nail hypoplasia, ectopic posterior pituitary
*RNPC3*	AR	CPHD	GH/TSH deficiency, cataracts, developmental delay, intellectual disability,
*LAMB2*	AR	GHD (syndromic)	Renal dysfunction; optic nerve and anterior pituitary hypoplasia
*TCF7L1*	AD (variable)	IGHD	Low IGF-1, isolated GHD
*RAX*	AR	CPHD	Anophthalmia, cleft lip/palate, absent pituitary
*POU1F1*	AR or AD	IGHD → CPHD	GHD + TSH and prolactin deficiency; highly variable onset and phenotype
*PROP1*	AR	IGHD → CPHD	GH, prolactin, TSH ± LH/FSH ± ACTH deficiency; micropenis
*IGSF1*	X-linked	IGHD (partial CPHD)	Central hypothyroidism, macroorchidism, delayed puberty
*ZBTB20*	AD (variable)	IGHD	Proportionate short stature, delayed growth
*KCNQ1*	AR or AD	GHD	Short stature, insulin and growth hormone disfunction, hearing loss
*DAX1*	X-linked	CGHD	Hypogonadotropic hypogonadism, hypothalamic–pituitary dysfunction, short stature

AD: autosomal dominant; AR: autosomal recessive; ACTH: adrenocorticotropic hormone; LH: luteinizing hormone; FSH: follicle-stimulating hormone; ADH: antidiuretic hormone; CNS: central nervous system; CPHD: combined pituitary hormone deficiency; GH: growth hormone; GI: gastrointestinal; IGHD: isolated growth hormone deficiency; GHD: growth hormone deficiency; TSH: thyroid-stimulating hormone; VSD: ventricular septal defect.

**Table 3:** Genes related to growth hormone insensitivity.

Gene	Inheritance	Syndrome/Medical Condition	Clinical and Labwork Features
*GHR*	AR or AD	Laron syndrome	Proportionate short stature, midface hypoplasia, frontal bossing, normal/elevated GH, low IGF-1, IGFBP-3, and ALS
*STAT5B*	AR or AD	GHI with immune dysfunction	Postnatal growth failure, immune dysregulation, pulmonary fibrosis, delayed puberty, elevated GH, low IGF-1
*STAT3*	AD	Autoimmune syndrome with growth failure	Short stature, multisystem autoimmunity (e.g., type 1 diabetes), impaired STAT1/STAT5 signaling
*IKBKB*	AD	GHI with NF-κB pathway defects	Growth retardation, partial GH/IGF-1 insensitivity, immune dysfunction
*IL2RG*	X-linked recessive	X-linked SCID with GHI	Severe IGF-1 resistance, poor GH response, immune deficiency
*PIK3R1*	AD	“SHORT” syndrome	Short stature, joint hyperextensibility, ocular depression, Rieger anomaly, teething delay, immune dysfunction

AD: autosomal dominant; AR: autosomal recessive; ALS: acid labile subunit; GH: growth hormone; GHI: growth hormone insensitivity; IGF-1: insulin-like growth factor 1; IGFBP-3: IGF-binding protein 3; SCID: severe combined immune deficiency; SHORT: short stature, joint hyperextensibility, ocular depression, Rieger anomaly, teething delay.

**Table 4:** Genes related to insulin-like growth factor 1-pathway disorders.

Gene	Inheritance	IGF Pathway Alteration	Clinical and Labwork Features
*IGF1*	AR (homozygous or compound heterozygous)	IGF-1 synthesis defect	Severe pre/postnatal growth failure, microcephaly, developmental delay, hearing loss, low IGF-1 with normal IGFBP-3/ALS
*IGF2*	Pathogenic variants are paternally inherited	IGF-2 synthesis defect	Growth restriction, dysmorphic features, confirmed link with Silver–Russell syndrome
*IGFALS*	AR	Ternary complex defect	Short stature, delayed puberty, hyperinsulinism, low IGF-1/IGFBP-3/ALS
*PAPPA2*	AR	Ternary complex defect	Short stature, microcephaly, skeletal anomalies, elevated total IGF-1, IGFBP-3, ALS; low free IGF-1
*STC2*	Only experimental data	Ternary complex regulator	Mouse studies: overexpression leads to short stature; loss-of-function mutations lead to tall stature
*IGF1R*	AD or AR (heterozygous, compound heterozygous)	IGF-1 resistance	Severe pre/postnatal growth failure, microcephaly, elevated IGF-1, poor response to GH therapy

AD: autosomal dominant; AR: autosomal recessive; GH: growth hormone; IGF-1: insulin-like growth factor 1; IGFBP-3: IGF-binding protein 3; ALS: acid labile subunit.
